# Targeting synthetic lethality between the SRC kinase and the EPHB6 receptor may benefit cancer treatment

**DOI:** 10.18632/oncotarget.10569

**Published:** 2016-07-13

**Authors:** James M. Paul, Behzad Toosi, Frederick S. Vizeacoumar, Kalpana Kalyanasundaram Bhanumathy, Yue Li, Courtney Gerger, Amr El Zawily, Tanya Freywald, Deborah H. Anderson, Darrell Mousseau, Rani Kanthan, Zhaolei Zhang, Franco J. Vizeacoumar, Andrew Freywald

**Affiliations:** ^1^ Department of Biochemistry, University of Saskatchewan, Saskatoon, SK, S7N 5E5, Canada; ^2^ Department of Pathology and Laboratory Medicine, College of Medicine, University of Saskatchewan, Royal University Hospital, Saskatoon, SK, S7N 0W8, Canada; ^3^ Department of Computer Science, University of Toronto, Toronto, ON, M5S 3G4, Canada; ^4^ The Donnelly Centre, University of Toronto, Toronto, ON, M5S 3E1, Canada; ^5^ Present address: Computer Science and Artificial Intelligence Laboratory, Massachusetts Institute of Technology, Cambridge, MA, 02139, USA; ^6^ Faculty of Science, Damanhour University, Damanhour, 22516, Egypt; ^7^ Cancer Research, Saskatchewan Cancer Agency, Saskatoon, SK, S7N 5E5, Canada; ^8^ Cell Signaling Laboratory, Neuroscience Cluster, University of Saskatchewan, Saskatoon, SK, S7N 5E5, Canada

**Keywords:** breast cancer, genetic interaction, synthetic lethality, EPHB6, SRC kinase

## Abstract

Application of tumor genome sequencing has identified numerous loss-of-function alterations in cancer cells. While these alterations are difficult to target using direct interventions, they may be attacked with the help of the synthetic lethality (SL) approach. In this approach, inhibition of one gene causes lethality only when another gene is also completely or partially inactivated. The EPHB6 receptor tyrosine kinase has been shown to have anti-malignant properties and to be downregulated in multiple cancers, which makes it a very attractive target for SL applications. In our work, we used a genome-wide SL screen combined with expression and interaction network analyses, and identified the SRC kinase as a SL partner of EPHB6 in triple-negative breast cancer (TNBC) cells. Our experiments also reveal that this SL interaction can be targeted by small molecule SRC inhibitors, SU6656 and KX2-391, and can be used to improve elimination of human TNBC tumors in a xenograft model. Our observations are of potential practical importance, since TNBC is an aggressive heterogeneous malignancy with a very high rate of patient mortality due to the lack of targeted therapies, and our work indicates that FDA-approved SRC inhibitors may potentially be used in a personalized manner for treating patients with EPHB6-deficient TNBC. Our findings are also of a general interest, as EPHB6 is downregulated in multiple malignancies and our data serve as a proof of principle that EPHB6 deficiency may be targeted by small molecule inhibitors in the SL approach.

## INTRODUCTION

The establishment of the estrogen receptor and human epidermal growth factor receptor-2 (HER2) as therapeutically relevant targets marked the development of genotype-directed treatment for breast cancer patients. The initial success in inhibiting key oncogenic drivers has stimulated extensive tumor genome sequencing aiming to identify genetic alterations for developing novel personalized therapies [1]. These personalized therapies targeting oncogenic alterations within a specific tumor are associated with minimal non-specific toxicity in cancer patients. Interestingly, tumor genome sequencing has also revealed numerous non-druggable genetic alterations such as deep deletions or epigenetic silencing in cancer cells. Development of mechanisms or tools to efficiently utilize these loss-of-function alterations for therapeutic purposes would dramatically expand our options in treatment personalization. In this context, the identification of synthetic lethal (SL) interactions, where suppression of one gene causes lethality only when another gene is also inactivated [2, 3], provides a unique opportunity to target these loss-of-function genetic defects. To exploit this model, we studied an unusual member of the Eph group of receptor tyrosine kinases (RTKs), the EPHB6 receptor. EPHB6 lacks catalytic activity due to several intrinsic alterations in the sequence of its kinase domain [4] and in contrast to other Eph receptors [5–7], EPHB6 is often downregulated in various malignancies, including metastatic lung cancer [8], melanoma [9], prostate cancer [10], ovarian carcinoma [11], gastric cancer [12], aggressive neuroblastoma [13, 14], and invasive breast cancer cell lines [15, 16]. This agrees well with the previously reported EPHB6 ability to suppress metastasis in non-small cell lung cancer [17] and melanoma [18], and our own findings that EPHB6 actively reduces breast cancer invasiveness [19]. Taken together, these observations indicate that EPHB6 receptor deficiency may potentially be targeted by using the SL approach to further personalize cancer therapy and improve treatment in multiple malignancies.

Triple-negative breast tumors represent a breast cancer subtype that is characterized by the lack of estrogen receptor (ER) and progesterone receptor (PR) expression and does not overexpress the HER2 receptor. Triple-negative breast cancer (TNBC) is associated with a very high rate of patient mortality due to the complete absence of targeted therapies and there is an active search for efficient therapeutic targets that would allow treatment personalization in TNBC tumors [20]. Here, we use a genome-wide shRNA-based screen and a xenograft model of human TNBC to assess a possibility that EPHB6 deficiency may be targeted in TNBC by the SL approach and examine if SL may assist in personalizing TNBC therapy.

## RESULTS

### Genome-wide shRNA screen reveals synthetic lethal interactions of the EPHB6 receptor in TNBC cells

**Figure 1:**
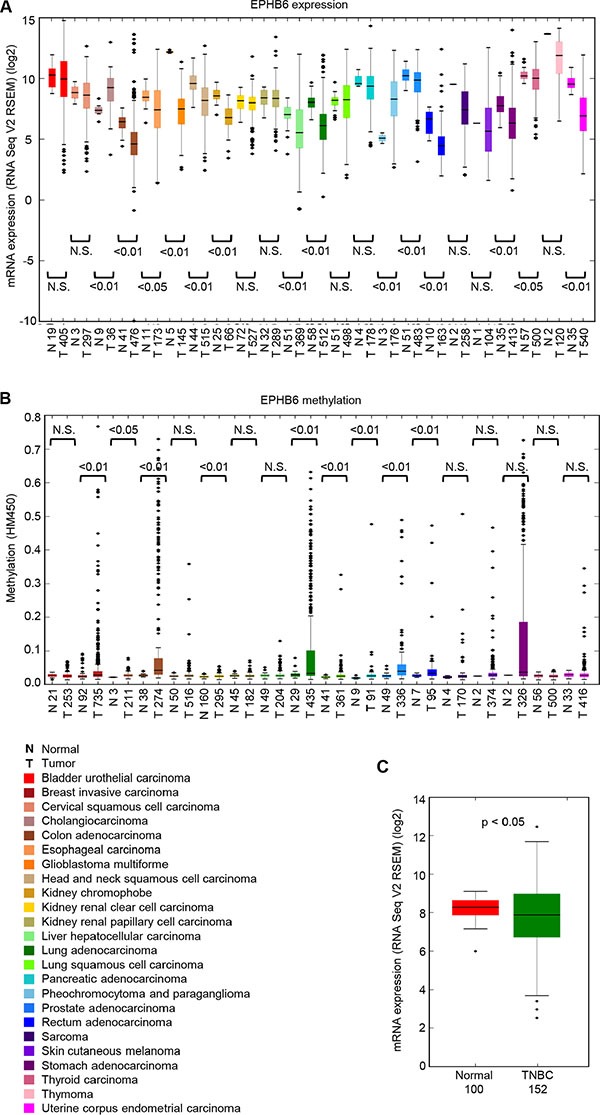
EPHB6 is downregulated in multiple human malignancies (**A**) EPHB6 expression was analyzed in twenty-three different cancer types and matching normal tissue controls using data from The Cancer Genome Atlas (TCGA) (cancergenome.nih.gov). The number of samples analyzed is shown on the x-axis. (**B**) Analysis of EPHB6 promoter methylation in eighteen different cancer types and matching normal tissue controls using data from TCGA. The number of samples analyzed is shown on the x-axis. The best methylation site was taken following the pre-processing step as outlined by the Broad Institute. The legend for panels (A) and (B) is presented below panel (B). (**C**) EPHB6 expression in normal and TNBC samples. TNBC samples were identified in the TCGA dataset based on the immunohistochemistry test. Statistical significance was computed using the Mann-Whitney *U* test.

A systematic analysis of the gene expression data from The Cancer Genome Atlas (TCGA) dataset (https://tcga-data.nci.nih.gov/tcga/) expanded previous observations and confirmed that EPHB6 is indeed downregulated in multiple tumor types (Figure 1A). As transcriptional regulation of EPHB6 was suggested to be controlled by promoter methylation in breast cancer cell lines [16], we analyzed human cancer methylome data and found that EPHB6 is methylated in the promoter region in several malignancies, including breast, colon, lung and prostate tumors (Figure 1B). Our assessment of TCGA data for immunohistochemistry-based breast cancer subtype classification revealed that EPHB6 is also significantly downregulated in patient samples, representing very heterogeneous and aggressive tumours of the TNBC group (Figure 1C). Further computational analysis based on immunohistochemistry data confirmed that EPHB6 expression is reduced in at least 60% of the TNBC tumours, when compared to its mean expression level in the matching normal tissue.

**Figure 2:**
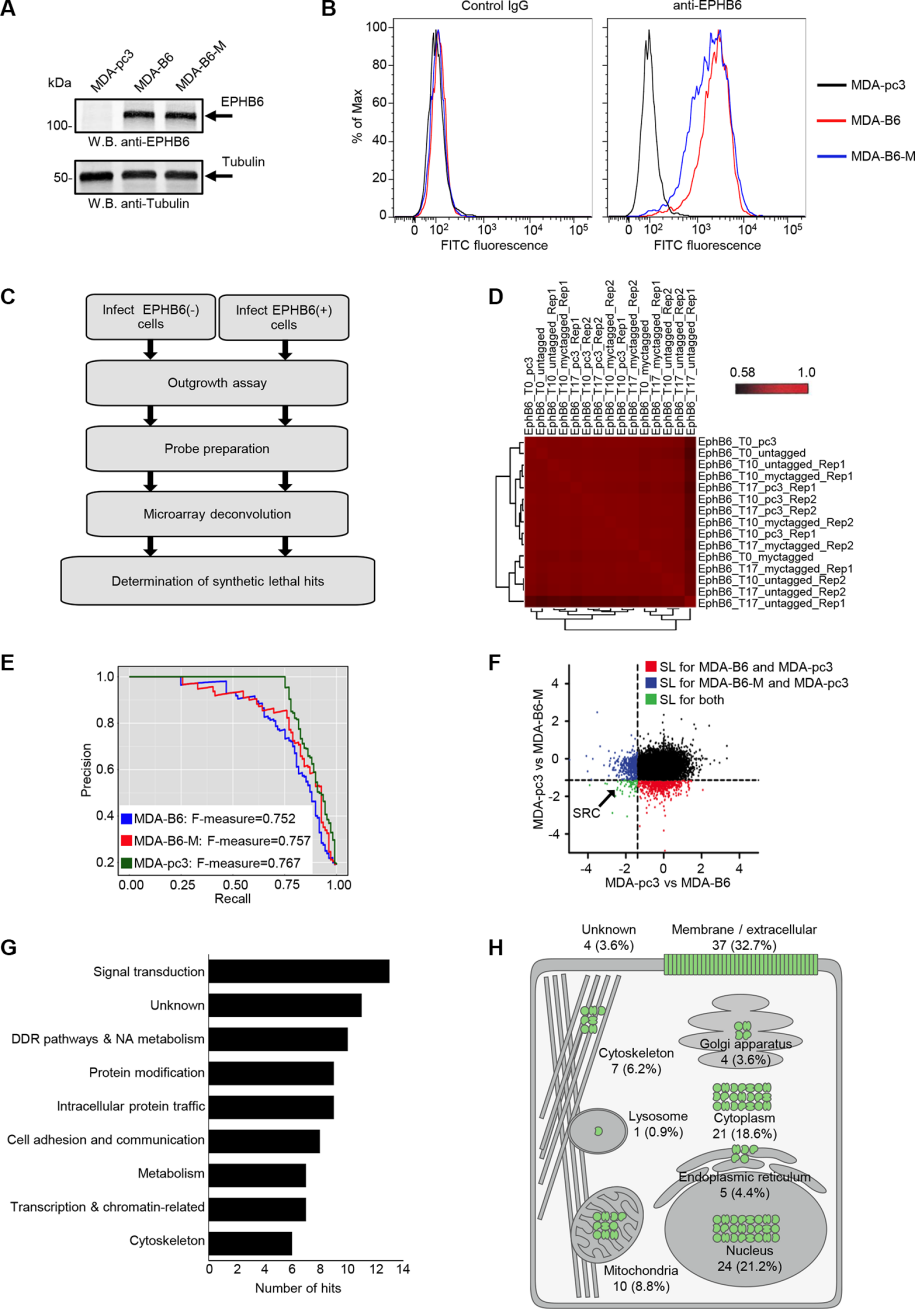
Genome-wide SL screen of EPHB6 (**A**) EPHB6 expression in EPHB6-deficient triple-negative breast cancer cells, MDA-MB-231, stably transfected with the pcDNA3 expression vector encoding wild-type EPHB6 (MDA-B6), myc-tagged EPHB6 (MDA-B6-M), or mock-transfected with empty pcDNA3 (MDA-pc3) was examined by Western blotting with anti-EPHB6. Western blotting with anti-tubulin was used as a loading control. (**B)** MDA-pc3, MDA-B6, and MDA-B6-M cells were stained with anti-EPHB6 and a FITC-conjugated secondary antibody, and analyzed by flow cytometry. Matching non-specific IgG was used as a control (Control IgG). (**C)** Schematic showing the steps of the shRNA pooled screening pipeline. (**D)** Pearson correlation between replicates of the pooled screen are clustered using hierarchical clustering with complete linkage. (**E)** Precision (TP/(TP + FP)) recall (TP/(TP + FN)) curve measuring the core essential and non-essential genes from the EPHB6 pooled screen. (**F)** Scatter plot showing the DCC score for every gene when MDA-pc3 is compared to both MDA-B6 and MDA-B6-M. (**G)** Analysis showing Gene Ontology terms associated with each screen. (**H)** Expected cellular distribution of EPHB6 synthetic lethal partners according to the Compartments Subcellular Localization Database (compartments.jensenlab.org).

Since there is a strong need for a targeted therapy in TNBC, we conducted our SL screens in well-characterized TNBC cells, MDA-MB-231, that are often used in breast cancer-related research [21, 22]. MDA-MB-231 represent an excellent model for our investigation, as the *ephb6* promoter is methylated and EPHB6 receptor expression is missing in these cells [15, 16]. In our experiments, we used cells with restored EPHB6 expression achieved by transfecting MDA-MB-231 cells with the pcDNA3 expression vector encoding wild-type EPHB6 (MDA-B6) or Myc-tagged EPHB6 (MDA-B6-M). Transfection with the empty vector was used as a control (MDA-pc3) (Figure 2A). These cells were described in our previous work [19]. Appropriate expression of the EPHB6 receptor on the surface of MDA-B6 and MDA-B6-M cells was confirmed by flow cytometry (Figure 2B).

We used a lentiviral library that contains 90,000 unique viral hairpins representing 18,000 human genes to analyze thousands of di-genic interactions across three genetic backgrounds (MDA-pc3, MDA-B6 and MDA-B6-M) in duplicates. Following the infection of our cell lines, gene knockdowns that caused lethality were identified by the loss of associated barcodes on microarrays (Figure 2C). The abundance of each shRNA was quantified by amplifying the hairpin sequences from the genomic DNA as a single mixture using vector-backbone directed universal primers. Specifically, shRNAs that dropped out in MDA-pc3, but not in MDA-B6 and MDA-B6-M populations are expected to target genes SL with EPHB6 deficiency. A correlation clustergram and the density plots of the three screens (MDA-pc3, MDA-B6, and MDA-B6-M) showed high reproducibility among the replicates (Figure 2D). This is because genetic interactions are rare [23], and the relatively high correlation between the replicates at the different time points even after considering gene drop out suggests that a few highly sensitive SL interactions were detected in our screens (Supplementary Figure S1A and S1B). Recently, a framework was developed for evaluating the quality of genome-scale lethality screens by assembling a reference set of essential genes [24]. If a high recall of these “gold standard” reference set of essential genes was achieved then the screen should be considered to be highly reliable [24]. Using this yardstick, we found that all three screens recorded excellent performance scores (F-measure > 0.7) (Figure 2E). In this analysis, the F-measure directly correlates with screen performance [24]. The trend of the hairpins that dropped specifically in EPHB6-deficient cells at different time points were computed as the Difference of Cumulative Change (DCC score) to identify top hits. The use of the top two hairpin scores per gene increased the confidence of the SL hits and allowed avoidance of possible off-target effects. As we used both Myc-tagged and untagged versions of EPHB6 in EPHB6-positive cells to compare against MDA-pc3, we determined the overlap between these two independent screens and identified 113 statistically significant overlapping hits (*p* < 0.05) (Figure 2F) (Supplementary Table S1). This level of overlap reflects the genomic instability of breast cancer cells and a rate of potential false positive hits associated with large-scale screens. Therefore, considering hits identified in two independent cell lines increased the confidence in our analysis. Our approach identified a number of potential candidates that predominantly function in signal transduction (Figure 2G), including molecules such as DDR2, SRC, ROCK2 and MET (Supplementary Table S1). Consistent with the receptor functions of EPHB6, cellular localization analysis of the hits also revealed that a significant percentage of SL molecules spatially associated with the cell surface (Figure 2H). Some of the hits were also associated with other cellular compartments, including nucleus and cytoplasm (Figure 2H), which reflected the complexity of the network of EPHB6 functional interactions in cancer cells.

**Figure 3:**
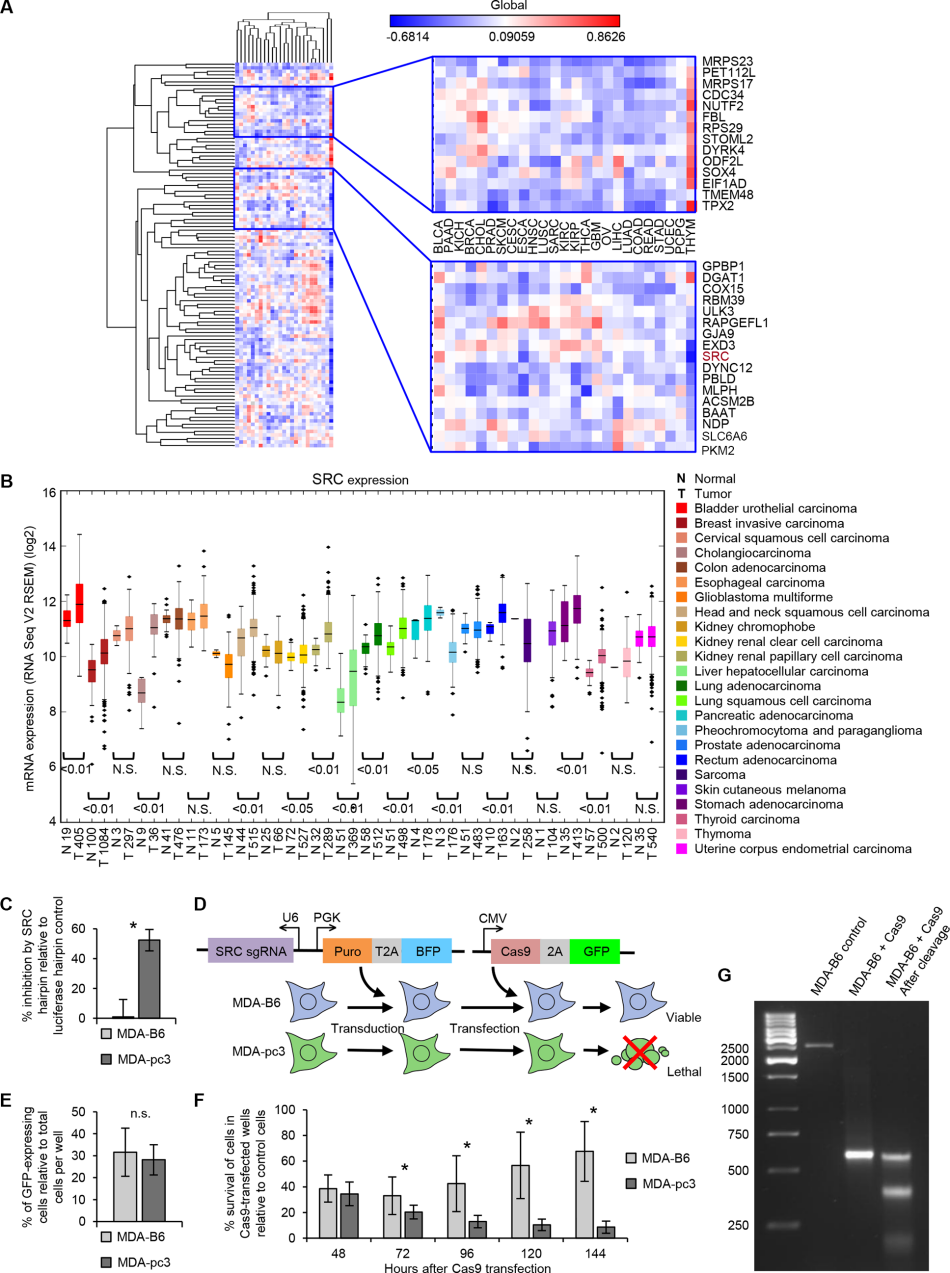
SRC is identified as a SL interacting partner of EPHB6 (**A**) Correlation clustergram showing expression of synthetic lethal hits (vertical) relative to EPHB6 expression (horizontal) across human malignancies. Negative correlations are blue and positive correlations are red. (**B**) Expression analysis of SRC in twenty-four different cancer types and normal tissue controls using data from TCGA. The number of samples analyzed is shown on the x-axis. (**C**) MDA-pc3 and MDA-B6 cells were transduced with SRC-targeting shRNA or luciferase-targeting shRNA as a control, and cultured in 96-well plates for 96 hours after puromycin selection. Cells were stained with resazurin and fluorescence was measured using a SpectraMax M5 microplate reader to determine cell suppression. The graph represents percentage of cell suppression by SRC hairpin relative to matching luciferase hairpin controls. Five wells were analyzed per condition. Experiment was performed three times. *P < 0.05, Student's *t*-test. (**D**) Schematic representation of the CRISPR/Cas9 strategy to validate the SL interaction. Cells of interest are stably transduced with a construct encoding *src*-targeting sgRNA and blue fluorescent protein (BFP), followed by the selection in the presence of 2 μg/mL of puromycin. The selected cells are transiently transfected with a construct encoding Cas9-2A-GFP. (**E**) MDA-pc3 and MDA-B6 cells were stably transduced with the src-targeting sgRNA construct that also encoded the blue fluorescent protein (BFP) and selected in the presence of 2 mg/ml of puromycin. The selected cells were transiently transfected with Cas9-GFP in 96-well plates and consistent transfection efficiency was confirmed by quantifying cells with green and blue fluorescence using the ImageXpress Micro XLS widefield automated fluorescence microscope and the MetaXpress version 6 software. The graph represents percentage of cells co-expressing Cas9-GFP and BFP relative to total cell numbers per well. (**F**) Surviving transfected cells from (E) were quantified at the indicated time points with the ImageXpress Micro XLS microscope and the MetaXpress software. The graph represents survival of transfected cells as a percentage relative to numbers of matching control cells expressing sgRNA/BFP only. Normalization on control cells was performed to account for a potential difference in proliferation rates of MDA-pc3 and MDA-B6 cells. In (E) and (F) each graph represents two independent experiments. At least ten wells were analyzed per condition in each experiment. *P < 0.05, Student's *t*-test. n.s., statistically not significant. (**G**) PCR amplification of *src*-sgRNA targeted genomic regions (with and without Cas9 expression) and DNA cleavage by the Detection Enzyme (GeneArt Genomic cleavage detection kit) are shown to demonstrate knockout of *src*.

We next attempted to prioritize a potential target for further validation from our screen. To systematically select potential candidates for further investigation, we undertook a novel approach, where we coupled SL data with gene expression profiles. We rationalized that increased expression of a SL gene in EPHB6-deficient cells most likely represents an essential compensatory mechanism. To identify these essential molecules, we compiled the correlation between EPHB6 expression and expression of each SL hit that was identified in the pooled shRNA screen. This analysis was done across 25 different tumor types and specifically searched for a negative correlation between expression of EPHB6 and a SL gene. Remarkably, we found a non-receptor tyrosine kinase, SRC, to be clustered with a set of genes that mostly correlated negatively with EPHB6 expression (Figure 3A). Consistent with this finding and in contrast to EPHB6 behavior, SRC is overexpressed in multiple malignancies (Figure 3B). In addition, functional network analysis of all the 113 hits obtained from the screen using the STRING 10 database (http://string-db.org), which quantitatively integrates genomic and previously published interactions, positioned SRC as a hub with high connectivity to the rest of the hits (Supplementary Figure S1C). Overall, these observations identified SRC as a molecule of choice for targeting EPHB6-deficient breast cancer cells.

To validate SL properties of SRC in EPHB6-deficient cells, we used an individual hairpin that efficiently silenced SRC expression (Supplementary Figure S1D). In agreement with our SL screen, we found that silencing of SRC with this hairpin caused a preferential suppression of EPHB6-deficient cells (Figure 3C). To completely exclude the involvement of potential off-target effects of shRNA molecules, we chose to also validate this SL interaction using the CRISPR/Cas9-based system (Figure 3D). Consistent with our earlier observations with SRC-silencing shRNAs, knockout of *src* with the CRISPR/Cas9 approach mostly affected EPHB6-defficient MDA-pc3 cells and produced only a limited effect on MDA-B6, thus further confirming the SL interaction between EPHB6 and SRC (Figure 3E–3G) (Supplementary Figure S2A).

### Synthetic lethal interaction between the SRC kinase and EPHB6 may be targeted by small molecule inhibitors in TNBC cells

**Figure 4:**
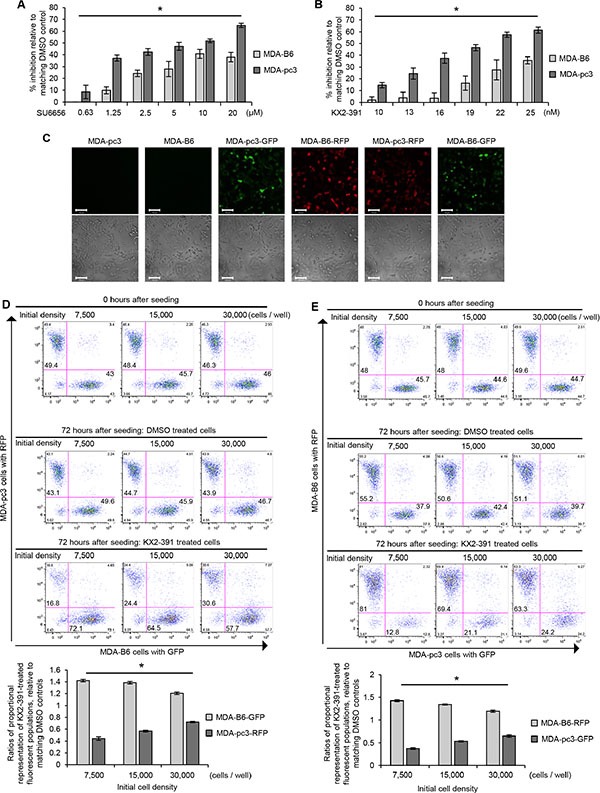
Validation of SL interaction between EPHB6 and SRC (**A**) MDA-pc3 and MDA-B6 cells were cultured in 96-well plates with the indicated concentrations of SU6656 or matching volumes of DMSO for 72 h. Cells were stained with resazurin and fluorescence was measured using a SpectraMax M5 plate reader to determine cell suppression. Five wells were analyzed per condition. The graph shows percentage of cell inhibition relative to DMSO control. (**B**) MDA-pc3 and MDA-B6 cells were cultured in 96-well plates with the indicated concentrations of KX2-391 or matching volumes of DMSO for 72 h. Cells were stained with resazurin and fluorescence was measured using a SpectraMax M5 plate reader to determine cell suppression. Five wells were analyzed per condition. The graph shows percentage of cell inhibition relative to DMSO control. (**C**) MDA-B6 and MDA-pc3 cells were transduced with lentivirus encoding pLD-GFP-Puro or pLD-RFP-Puro as indicated. Cells were selected with 2 μg/mL puromycin for 48 h, cultured in monolayer, and imaged by confocal microscopy at 100 x magnification. (**D**) GFP-expressing MDA-B6 cells (MDA-B6-GFP) and RFP-expressing MDA-pc3 (MDA-pc3-RFP) were combined in equal numbers at the indicated cell densities in 24-well plates, and cultured with 25 nM KX2-391 or DMSO for 72 h. Cells were collected and analyzed by flow cytometry and the FlowJo software. The graph represents analysis of triplicates and shows ratios of proportional representation of KX2-391-treated fluorescent populations relative to matching DMSO controls. (**E**) RFP-expressing MDA-B6 (MDA-B6-RFP) and GFP-expressing MDA-pc3 (MDA-pc3-GFP) cells were combined in equal numbers at the indicated cell densities in 24-well plates and cultured with 25 nM KX2-391 or matching volume of DMSO for 72 h. Cells were collected and analyzed by flow cytometry and the FlowJo software. The graph represents analysis of triplicates and shows ratios of proportional representation of KX2-391-treated fluorescent populations relative to matching DMSO controls. All experiments were performed at least three times. Scale bar, 100 μM. *P < 0.05, Student's *t*-test.

As SRC plays an important role in breast cancer progression and several SRC inhibitors are already being tested in breast cancer clinical trials [25], we used SRC inhibitors to further assess its SL properties. To model the SL interaction observed between SRC and EPHB6 by chemical genetics, we treated MDA-pc3 and MDA-B6 cells with increasing concentrations of SRC inhibitor, SU6656. Consistent with the effects of the SRC-targeting shRNA or *src* knockout (Figure 3C and 3F), application of SU6656 preferentially suppressed EPHB6-deficient MDA-pc3 cells (Figure 4A). Another SRC inhibitor, KX2-391, has been tested in phase II clinical trials for prostate cancer treatment, where it showed a relatively modest effect [26]. KX2-391 is currently also being tested for breast cancer treatment (NCT01764087) and our finding of the SL relationship between EPHB6 and SRC indicated that KX2-391 treatment may work more efficiently if applied specifically to EPHB6-deficient TNBC cells. To assess this possibility, we incubated MDA-pc3 and MDA-B6 cells with this inhibitor or matching solvent control. In similarity to SU6656 action, KX2-391 caused significantly stronger suppression of EPHB6-deficent cells (Figure 4B), suggesting that KX2-391 treatment may indeed potentially benefit from a more personalized approach, where it would be applied exclusively to EPHB6-deficient tumors. This important observation was further confirmed in experiments with co-cultured MDA-B6 and MDA-pc3 cells, which allowed us to exclude any influence of potential differences in tissue culture conditions on experimental outcomes. In this model, EPHB6-defficient and EPHB6-expressing cells were stably transduced with a lentiviral vector expressing green or red fluorescent proteins (Figure 4C). Cells were mixed, co-seeded in equal numbers, treated with KX2-391 or solvent control and cell suppression was monitored by flow cytometry. These experiments also clearly showed that EPHB6-deficient cells are much less resistant to SRC inhibition (Figure 4D and 4E).

**Figure 5:**
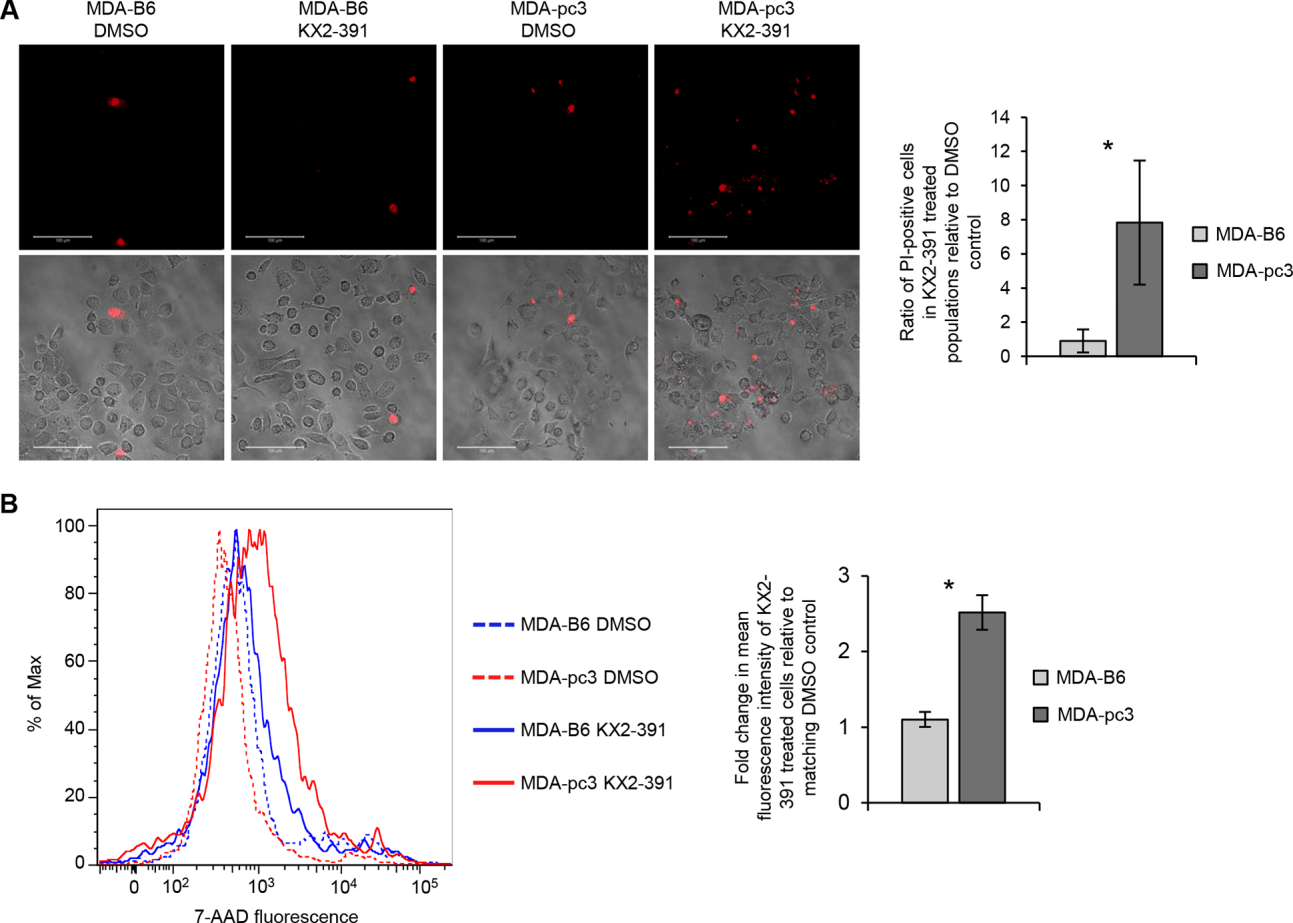
Inhibition of SRC induces cell death more efficiently in EPHB6-deficient cells (**A**) MDA-pc3, and MDA-B6 cells were cultured in glass-bottom plates in the presence of 25 nM KX2-391 or DMSO for 72 h and stained with 2.7 μg/mL propidium iodide (PI) in phenol red-free medium. Stained cells were imaged at 200 x magnification using Zeiss LSM 700 confocal microscope and PI-stained cells were counted in at least 10 randomly captured frames. Counts of PI-positive cells were normalized on the total cell numbers in matching frames. The graph shows the ratio of PI-positive cells in KX2-391-treated populations relative to matching DMSO controls. (**B)** MDA-pc3 and MDA-B6 cells were cultured in 6-well plates in the presence of 25 nM KX2-391 or a matching volume of DMSO for 72 h. Cells were collected and stained for 7-AAD for 15 minutes at room temperature. Cells were analyzed by flow cytometry and the FlowJo software. The graph represents analysis of triplicates and shows fold change in mean fluorescence intensity in KX2-391-treated cells relative to matching DMSO controls. All experiments were performed at least three times. Scale bar, 100 μm. *P < 0.05, Student's *t*-test.

To examine if preferential suppression of EPHB6-defficient cells observed in our experiments is associated with more efficient cell killing, we exposed KX2-391-treated cultures to Propidium Iodide or 7-AAD compounds that stain nonviable cells only. Both approaches revealed that KX2-391 is more efficient in inducing cell death, when the EPHB6 receptor is not expressed (Figure 5A and 5B).

**Figure 6:**
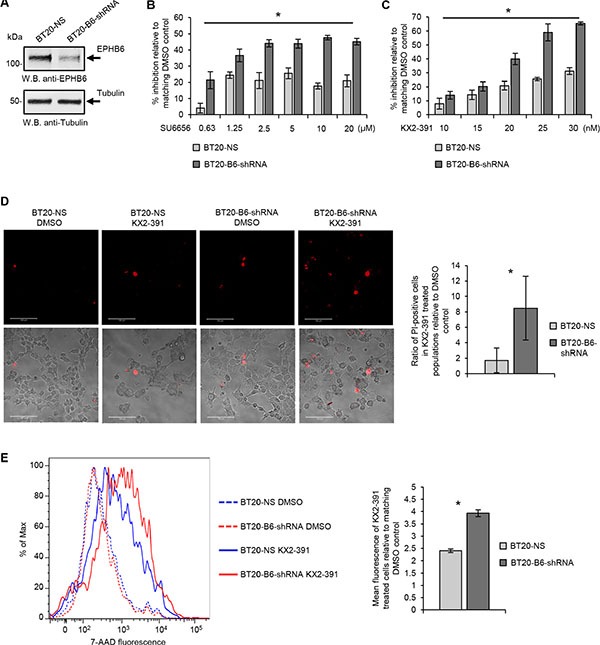
Validation of SL relation between EPHB6 and SRC in BT-20 TNBC cells (**A**) Triple negative breast cancer cells, BT-20, were transduced with EPHB6-targeting shRNA (BT20-B6-shRNA), or non-silencing shRNA (BT20-NS). EPHB6 expression was analyzed by Western blotting with anti-EPHB6. Western blotting with anti-tubulin was used as a loading control. (**B)** BT20-B6-shRNA and BT20-NS cells were cultured in 96-well plates with indicated concentrations of SU6656 or matching volumes of DMSO for 96 h. Cells were stained with resazurin and fluorescence was measured using a SpectraMax M5 plate reader to determine cell suppression. Five wells were analyzed per condition. The graph shows percentage of cell suppression relative to DMSO control. **(C)** Cells were cultured in 96-well plates with indicated concentrations of KX2-391 or matching volumes of DMSO for 96 h. Cells were stained with resazurin and fluorescence was measured using a SpectraMax M5 plate reader to determine cell suppression. Five wells were analyzed per condition. The graph shows percentage of cell suppression relative to DMSO control. (**D)** BT20-B6-shRNA and BT20-NS were cultured in glass-bottom plates in the presence of 35 nM KX2-391 or DMSO for 96 h. Cells were stained with 2.7 μg/mL propidium iodide (PI) in phenol red-free medium and imaged using confocal microscopy. PI-stained cells were counted in at least 10 randomly captured frames. Counts of PI-positive cells were normalized on the total cell numbers in matching frames. The graph shows the ratio of PI-positive cells in KX2-391-treated populations relative to matching DMSO controls. (**E)** Cells were cultured in 6-well plates in the presence of 25 nM KX2-391 or DMSO for 96 h, collected and stained with 7-AAD. Stained cells were analyzed by flow cytometry and the FlowJo software. The graph represents analysis of triplicates and shows the mean fluorescence intensity of KX2-391-treated cells relative to DMSO control. All experiments were performed at least three times. Scale bar, 100 μM. *P < 0.05, Student's *t*-test.

Importantly, the SL interaction between EPHB6 and SRC observed in our work was not restricted to MDA-MB-231 cells, since silencing of EPHB6 expression in another TNBC cell line, BT-20, strongly increased their suppression by both SU6656 and KX2-391 (Figure 6A–6E).

Despite the SL relation between EPHB6 and SRC that we observed in our work, EPHB6 did not affect SRC inhibition, as SRC was efficiently inhibited by SU6656 in MDA-B6 cells (Supplementary Figure S2B). These observations suggest that EPHB6 makes TNBC cells more resistant to SRC inhibitors, not by interfering with their direct effects on SRC activity, but most likely by compensating for the loss of SRC action in cellular responses controlled by this molecule.

### The EPHB6-SRC synthetic lethality enhances suppression of TNBC tumors

**Figure 7:**
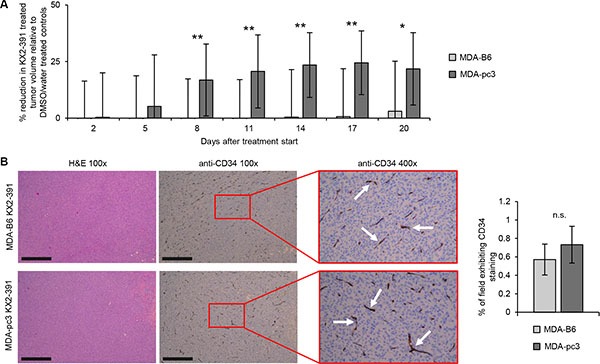
Analysis of the SL interaction between EPHB6 and SRC in TNBC cells and murine xenografts (**A**) MDA-pc3 and MDA-B6 cells were injected into the mammary fat pad region of 4–6 weeks old NOD-SCID mice (1 × 10^6^ cells per mouse). Mice with detectable tumors were treated twice per day with 5 mg/kg KX2-391 in DMSO/water solvent or solvent alone by oral feeding (at least 6 animals per each experimental condition). Tumor size was measured every 3 days and tumor volume was calculated with the equation: A/2*B^2^, where A was long and B was short diameter of the tumor. The reduction in tumor growth in KX2-391-treated mice is presented as a percentage relative to matching solvent controls. The graph summarizes two independent experiments. Day 0 indicates the beginning of treatment with KX2-391 or matching solvent control. The experiments were terminated upon tumor ulceration according to the guidelines established by the Animal Research Ethics Board, University of Saskatchewan. (**B)** KX2-391-treated MDA-B6 and MDA-pc3 tumors from (A) were extracted upon experiment termination, fixed in 10% neutral-buffered formalin, and paraffin embedded. Tumor sections were processed for immunohistochemical staining with anti-CD34 or stained with haematoxylin and eosin (H&E). Four representative fields (at 3, 6, 9, and 12 o'clock) per each stained tumor section (one for each extracted tumor) were imaged at 100 × magnification and the blood vessel density per each field was analyzed with the Image-Pro Premier software. The graph represents percentage of anti-CD34-positive area relative to the overall field of view. Images of representative areas highlighted by red rectangles are shown at 400 x magnification. Arrows indicate representative examples of anti-CD34-stained blood vessels. At least 6 stained sections per each experimental condition representing independent tumours were used for the analysis. Scale bar, 500 μM. *P < 0.05; **P < 0.01, Student's *t*-test. n.s., statistically not significant.

As SRC inhibitors are being actively evaluated in breast cancer clinical trials [25], our findings strongly suggested that the SL interaction between EPHB6 and SRC might be used to target TNBC tumors. To test this, we produced TNBC tumors in experimental animals by injecting MDA-pc3 and MDA-B6 cells in mammary fat pad regions of immunodeficient female NOD.Cg-*Prkdc^scid^ Il2rg^tm1Wjl^*/SzJ (NOD-SCID) mice. Treatment of these animals with KX2-391 was initiated when tumors reached a detectable size and was carried on until the mice had to be eliminated in accordance with the guidelines established by the University of Saskatchewan Animal Research Ethics Board. Excitingly, these experiments revealed that the KX2-391 therapy, indeed, more efficiently suppresses growth of EPHB6-deficient TNBC tumors (Figure 7A). Staining for a blood vessel marker, CD34, has not shown any differences in neovascularization of KX2-391-treated EPHB6-positive or EPHB6-negative tumors, confirming that the observed lower resistance of EPHB6-deficient tumors was not due to the preferential suppression of blood vessel formation, but because of the higher sensitivity of EPHB6-deficient TNBC cells (Figure 7B).

## DISCUSSION

SL interactions have opened a new avenue for developing targeted therapies and personalized medicine. For example, at least three clinical trials have been initiated using EGFR and BRAF inhibitors within three years after the SL relation between EGFR and BRAF has been identified [30] (NCT01791309; NCT01750918; NCT01719380). This rapid progress into clinical trials is triggered by selective focusing on well-studied targets with the FDA approved inhibitors. The genome-wide SL screens discussed here revealed a novel genetic interaction between the SRC kinase and EPHB6 in TNBC cells. Moreover, network assessment directly indicated that SRC is a central player with a high connectivity. Our expression analysis also showed that SRC clusters with the genes that negatively correlate with EPHB6 expression in various tumors. This indirectly suggested that SRC overexpression might act as an essential compensatory mechanism for the loss of EPHB6 in cancer cells, indicating that the SL interaction of EPHB6 and SRC may represent a promising therapeutic target. The SRC kinase inhibitor, KX2-391, is already being tested in clinical trials and our investigation provides a new rationale for the selective use of KX2-391 in patients that have lost expression of the EPHB6 receptor in their tumors. The relevance of this finding is further supported by recent unfortunate observations, revealing that although SRC is frequently overexpressed in cancer, in some clinical trials randomly applied SRC inhibition produced limited positive effects on cancer patients [26]. Our report of the SL relation between EPHB6 and SRC may help to overcome this problem, and improve the efficiency of SRC inhibiting approaches in cancer therapy by showing that treatment with SRC inhibitors should be personalized, and mostly applied to patients with reduced or missing EPHB6 expression. In this context, it is important that our analysis confirmed that the SL interaction between SRC and EPHB6 can be efficiently targeted by small molecule SRC inhibitors and revealed that EPHB6-deficient TNBC cells are, indeed, much more sensitive to these compounds. Our experimental data suggest that EPHB6 does not protect SRC from inhibition and we suspect that EPHB6 most probably acts by partially compensating for the loss of the biological functions of the SRC kinase. This also explains well the ability of EPHB6 to protect cancer cells from shRNA-induced silencing of SRC or *src* knockout observed in our work. This model fits a classical definition of a SL interaction [31] and provides a rationale for a limited effectiveness of SRC-inhibiting therapy currently observed in some cancer patients [26].

Consistent with the higher sensitivity of EPHB6-negative TNBC cells to SRC inhibition, an FDA-approved SRC kinase inhibitor, KX2-391, proved to be significantly more effective in suppressing EPHB6-deficient TNBC tumors, when compared to its effect on matching tumors with restored EPHB6 expression. These findings are of a potential practical importance, as our work reveals that although EPHB6 expression is overall downregulated in TNBC, it appears to be better preserved in a certain portion of TNBC tumors (Figure 1C). Our observations indicate that in this situation, EPHB6 may be efficiently used as a biomarker for selecting exclusively EPHB6-deficient TNBC tumors for the treatment with SRC inhibitors, while re-directing patients with high EPHB6 expression in their tumors for more appropriate therapeutic options. Such a personalized approach is likely to assure successful utilization of SRC-inhibiting therapies and would also benefit patients with EPHB6-positive TNBC by preventing their involvement in ineffective treatment protocols. This of course would require a further evaluation of EPHB6 function in freshly obtained tumour samples. Our model may also potentially be applicable to multiple other tumor types, where EPHB6 expression is reduced according to previously published observations [8–14] and according to our findings reported here. We plan to assess this possibility in our future work. As our investigation follows a genome-wide SL screen by a successful validation of its results in the animal model of human malignancy, it provides a strong support for a broad use of this methodology in finding new therapeutic targets and developing personalized treatment approaches.

## MATERIALS AND METHODS

### Ethics statement

Investigation has been conducted in accordance with the ethical standards and according to the Declaration of Helsinki and according to national and international guidelines and has been approved by the authors' institutional review board.

### Antibodies and reagents

Anti-phospho-SRC was from Life Technologies (Burlington, ON, Canada). Anti-c-SRC, anti-β-tubulin and SU6656 were from Santa Cruz Biotechnology (Dallas, TX, USA). Human anti-EPHB6 antibody, matching sheep IgG control, FITC-conjugated anti-sheep secondary antibody, and resazurin were from R&D Systems (Minneapolis, MN, USA). BSA was from BioShop Canada Inc. (Burlington, ON, Canada). KX2-391 was from Selleckchem (Houston, TX, USA). 7-AAD kit was from BD Biosciences (Mississauga, ON, Canada). Dimethyl sulfoxide (DMSO) and polybrene were from Sigma-Aldrich (St. Louis, MO, USA). Propidium iodide and puromycin were from ThermoFisher Scientific (Burlington, ON, Canada). Pooled screen shRNAs and constructs were derived from the RNAi Consortium lentiviral library (Sigma-Aldrich). sgRNA constructs encoding BFP were from MilliporeSigma/welcome trust Sanger (Sigma-Aldrich). pLD-GFP-puro and pLD-RFP-puro expression constructs were previously described [3]. The GeneArt Genomic cleavage detection kit was from ThermoFisher Scientific.

### Cell lines and culture conditions

MDA-MB-231 and BT-20 cells were purchased from the American Type Culture Collection (Manassas, VA, USA). Cells were passaged for less than three months at a time following resuscitations and therefore, no additional authentication was performed. Both MDA-MB-231 and BT-20 monolayer cultures were maintained in the DMEM medium containing 10% FBS (Gibco, Life Technologies), 1% penicillin/streptomycin (Gibco, Life Technologies) and 1 mM sodium pyruvate (HyClone, GE Life Sciences,).

### Stable cell lines and lentiviral transduction

Stable MDA-MB-231 cell lines with restored EPHB6 expression were generated by transfecting MDA-MB-231 cells with the pcDNA3 expression vector encoding wild-type EPHB6 (MDA-B6) or Myc-tagged EPHB6 (MDA-B6-M). Transfection with the empty vector was used as a control (MDA-pc3). Stable EPHB6 knockdowns were generated using EPHB6-targeting shRNA encoded in lentiviral particles (Santa Cruz Biotechnology, Dallas, TX, USA). Cells were transduced using 10 μg/mL polybrene (Sigma-Aldrich), followed by 5 days of selection with 10 μg/mL puromycin (Sigma-Aldrich). Transduction with SRC-targeting shRNA constructs and with GFP- or RFP-encoding cDNAs, required preparation of lentiviral particles. Lentiviral particles were generated by transfection of HEK-293T cells, grown in 10 cm plates to ~70% confluence with psPAX2, pMD2.G, and with the lentiviral vector encoding the genes of interest. Transfection took place in 10 mL of tissue culture medium with 1,400 μL Opti-Mem (Gibco, Life Technologies) and 93.6 μl X-treamGENE 9 DNA Transfection Reagent (Roche, Mississauga, ON, Canada). Medium was changed 18 hours later and replaced with DMEM containing 2% (w/v) bovine serum albumin (BSA) and viral particles were collected 48 h and 72 h after transfection. MDA-B6 and MDA-pc3 cells were transduced with the lentiviral particles by incubation overnight in medium containing 8 μg/mL polybrene. The transduction medium was removed and transduced cells were incubated for 48 h in cell culture medium containing 2 μg/mL puromycin.

### CRISPR/Cas9 analysis

MDA-B6 and MDA-pc3 cells were seeded in 6-well plates and transduced with *src*-targeting sgRNA lentiviral constructs that also encoded BFP in the presence of 8 μg/mL of polybrene. Following 48 h of selection with 2 μg/mL puromycin, selected cells were seeded in 96-well optical bottom plates (ThermoFisher Scientific), allowed to adhere for 24 h, and transfected with CMV-Cas9-2A-GFP (Sigma CAS9GFPP-1EA) using the Lipofectamine LTX and Plus Reagent kit (ThermoFisher Scientific) according to the manufacturer's instructions. Cells were imaged every 24 hours for six days after transfection using the ImageXpress Micro XLS widefield automated fluorescence microscope (Molecular Devices, Sunnyvale, CA, USA) to capture BFP and GFP signals. Cells expressing BFP or co-expressing BFP and Cas9-GFP were quantified using MetaXpress version 6 (Molecular Devices). *src* knockout was confirmed using the GeneArt Genomic cleavage detection kit (ThermoFisher Scientific) following the manufacturer's instructions.

### Drug sensitivity assays

MDA-MB-231 and BT-20 cell monolayers were incubated in 96-well plates for 72 h and 96 h, respectively, with indicated concentrations of KX2-391 or SU6656, or matching volumes of DMSO as a solvent control. Treated cells were stained using resazurin by following the manufacturer's instructions and fluorescence was measured using a SpectraMax M5 microplate reader.

### Western blotting

Cells were rinsed with ice-cold PBS and lysed using lysis buffer containing 0.1 M EDTA, 0.3 M Tris, 0.1 M NaCl, 6 mM PMSF, and 3 mM sodium ortho-vanadate. Cell debris were removed by centrifugation. For immunoprecipitation, 2–3 μg of required antibody, with 25 μL of protein G Sepharose beads (GE Healthcare Life Sciences, Baie d'Urfe, QB, Canada) were added. Samples were rotated at 4°C overnight and beads were washed three times with lysis buffer. Cell lysates or immunoprecipitates were resolved using SDS-PAGE, followed by transfer to nitrocellulose membranes (Amersham, GE Healthcare Life Sciences). Membranes were blocked with 5% non-fat dry milk in 0.1% PBS/Tween-20, or with 5% BSA in TBS/Tween-20 and incubated overnight with primary antibodies at 4°C. At this stage, membranes were rinsed 3 times with PBS or TBS, incubated for 1 h with fluorescently labeled secondary antibodies (LI-COR Biotechnology, Guelph, ON, Canada) and protein images were acquired using the LI-COR Odyssey imaging system (LI-COR Biotechnology). Figures were generated using the Odyssey, Carestream and PowerPoint software. Cropping of Western blot images was done with PowerPoint. Brightness and contrast were adjusted in western blot images using Carestream and Powerpoint software to optimize image presentation. Western blotting with anti-tubulin was used as a loading control.

### Drug sensitivity assays with fluorescent cells

For color assays, MDA-B6-GFP and MDA-pc3-RFP, or MDA-B6-RFP and MDA-pc3-GFP cells were co-seeded in equal numbers in 12-well plates at indicated cell densities. Seeded cells were incubated for 72 h with 25 nM KX2-391 and a matching volume of DMSO. Treated cells were collected and quantitated by flow cytometry. Results were analyzed using the FlowJo software (FLOWJO LLC, Ashland, OR, USA).

### Monitoring expression of EPHB6 on the cell surface

To confirm cell surface expression of EPHB6 in MDA-B6 and MDA-B6-M, cells were collected with 2 mM EDTA, washed with serum-free media, and incubated with anti-EPHB6 or matching IgG control for 40 minutes on ice. Labeled cells were washed twice with serum-free media, and incubated with FITC-conjugated secondary antibody for 30 minutes on ice in the dark. Cells were then washed twice with serum-free media and suspended in PBS for analysis by flow cytometry. Results were analyzed using the FlowJo software (FLOWJO LLC).

### Cell death assays

For propidium iodide (PI) staining, MDA-MB-231 and BT-20 cells were incubated in glass-bottom plates (MatTek, Ashland, MA, USA) for 72 h and 96 h, respectively, with KX2-391 and matching volumes of DMSO. Cells were then incubated with 2.7 μg/ml PI for 12 minutes and washed with phenol red-free medium. The amount of PI-stained cells was analyzed by microscopy using a Zeiss Observer Z1 at 200 x magnification. Brightness of presented confocal microscopy images was adjusted using the Zen 2012 Software (version 8.0) to optimize the visualization of PI staining. PI-stained cells were counted in at least 10 randomly captured frames, normalized on the total number of cells in matching frames and compared between DMSO controls and treated cells.

For 7-AAD staining, MDA-MB-231 and BT-20 cells were incubated in 6-well plates for 72 h and 96 h, respectively, with 25 nM KX2-391 and matching volumes of DMSO. Cells were collected and stained with 7-AAD according to the manufacturer's instructions, prior to flow cytometry analysis. Results were analyzed using the FlowJo software (FLOWJO LLC).

### Tumor xenograft studies and immunohistochemistry

Breeder pairs of NOD SCID gamma mice were purchased from The Jackson Laboratory and a colony was established at the Laboratory Animal Services Unit, University of Saskatchewan. Mice were housed in sterile cages and maintained in pathogen-free aseptic rooms, while being fed autoclaved food pellets and water *ad libitum*. All animal protocols were reviewed and approved by the University of Saskatchewan Animal Research Ethics Board. Xenograft tumors were established by injection of 1 × 10^6^ MDA-B6 or MDA-pc3 in 100 μL PBS into the mammary fat pads of 4–6 week old female animals. Treatments with KX2-391 were initiated when tumors became palpable. Mice were fed with either KX2-391 (5 mg/kg) in DMSO/water solvent or a matching volume of the solvent. Treatments were administered orally twice a day. Digital caliper measurements were taken every 3 days and tumor volume was calculated by the formula A/2*B^2^ (where A and B were the long and short diameters of the tumor respectively). At the end of the experiments animals were sacrificed and tumors were removed. Tumors were fixed in 10% buffered formalin for paraffin embedding.

For the immunohistochemical staining, tumors were dissected and fixed in 10% neutral- buffered formalin for 24–48 h. The tumors were paraffin embedded, sectioned to 4 μm thickness, and affixed on the slide. Simultaneous dewaxing and antigen retrieval was performed on the Dako PT Link using Target Retrieval Solution-High pH (Dako Canada, Burlington, ON, Canada). Staining was performed on the Dako Autostainer Link using anti-CD34 (Abcam, Toronto, ON, Canada) antibody and the Dako FLEX DAB+Detection Kit. In each stained tumor section, 12, 3, 6 and 9 o'clock fields were imaged at 100 × magnification and the density of stained blood vessels per field was quantified using the Image-Pro Premier software.

### Expression analysis

Expression data from TCGA datasets for different cancer types was collected in regard to both EPHB6 and SRC. The distribution was plotted for both tumor patients and normal patients. TCGA methylome data was also collected for EPHB6 and the distribution was plotted for both tumor patients and normal patients. Ovarian cancer that was analyzed in the correlation clustergram (Figure 3A) is not included in the expression analysis due to the unavailability of data in matching normal tissue.

### Pooled screening

Pooled shRNA screening was done as previously described [3]. Briefly, MDA-B6, MDA-B6-M, and MDA-pc3 cells were transduced with lentiviral particles containing a 90K shRNA library with ~200x hairpin representation. Cells were passaged for 17 days and genomic DNA was collected at T0, T10, and T17 for analysis. Genomic DNA was amplified by large-scale PCR. The amplification PCR reaction was carried out by denaturing once at 98°C for 3 minutes, followed by (98°C for 10 seconds, 55°C for 15 seconds, 72°C for 15 seconds) x29, 72°C for 5 minutes, then cooling to 4°C. Amplification products were purified and digested with XhoI (New England Biolabs, Whitby, ON, Canada). The stable half-hairpins were purified and probe hybridization was carried out on UT-GMAP 1.0 microarrays (Affymetrix Inc, Santa Clara, CA, USA).

### Computational scoring of pooled screens

For each hairpin, the signal intensity was normalized and converted to log2 scale for each time point of both MDA-wild type, and MDA-pc3 samples. Note that the MDA-wild type samples were either MDA-B6 or MDA-B6-M. Hairpins whose signal was below the background (i.e. log2 scale of less than 8) at time point T0 were discarded. Likewise, hairpins with fold-change greater than or equal to 1.25 at a time point relative to the corresponding previous time point were also discarded. For each replicate, the difference of cumulative change (DCC) between the MDA-pc3 and MDA-wild type conditions were calculated for time points relative to the corresponding previous time point using the formula:

$$DCC\;=\sum_{t=1}^{T}(x_{t,k}^{pc3}-x_{t-1,k}^{pc3})-\sum_{t=1}^{T}\left(x_{t,k}^{w}-x_{t-1,k}^{w}\right)$$

where $x_{t,k}^{pc3}$ is the normalized signal intensity at time point *t* ∈ (0,..,*T*) and for replicate *k* ∈ (1,..,*K*) for MDA-pc3 samples. Likewise, $x_{t,k}^{w}$ represent the same for MDA-B6 or MDA-B6-M samples. The DCC fitness score was then calculated for each gene by taking the two hairpin DCC values that were the most negative values for that gene.

$$DCC_{g}=arg_{h,h'}^{\min}[DCC_{g,h}+DCC_{g,h'}]/2$$

Next, the permutation test was performed by randomly shuffling the DCC scores. This process was repeated and an empirical distribution of the DCC fitness scores over all of the genes was constructed. Finally, significant *p*-values for each observed fitness score were estimated as the frequency of randomized, shuffled DCC with more negative scores.

$$p=\frac{1}{NL}\sum_{r=1}^{NL}I(DCC_{r}<DCC_{g})$$

where N is the number of genes, L is the number of repeats done to construct an empirical distribution, *DCC_r_* is the randomized shuffle with more negative score, and *I*() is a binary indicator that give 1 for a true statement, and 0 otherwise.

### Statistical analysis

Student's *t*-test was performed for statistical analyses, until otherwise indicated. Data are presented as mean ± SD.

## SUPPLEMENTARY MATERIALS FIGURES AND TABLES



## References

[1] Arnedos M, Vicier C, Loi S, Lefebvre C, Michiels S, Bonnefoi H, Andre F (2015). Precision medicine for metastatic breast cancer-limitations and solutions. Nat Rev Clin Oncol.

[2] Paul JM, Templeton SD, Baharani A, Freywald A, Vizeacoumar FJ (2014). Building high-resolution synthetic lethal networks: a ‘Google map’ of the cancer cell. Trends Mol Med.

[3] Vizeacoumar FJ, Arnold R, Vizeacoumar FS, Chandrashekhar M, Buzina A, Young JT, Kwan JH, Sayad A, Mero P, Lawo S, Tanaka H, Brown KR, Baryshnikova A (2013). A negative genetic interaction map in isogenic cancer cell lines reveals cancer cell vulnerabilities. Mol Syst Biol.

[4] Gurniak CB, Berg LJ (1996). A new member of the Eph family of receptors that lacks protein tyrosine kinase activity. Oncogene.

[5] Pasquale EB (2010). Eph receptors and ephrins in cancer: bidirectional signalling and beyond. Nat Rev Cancer.

[6] Lisabeth EM, Falivelli G, Pasquale EB (2013). Eph receptor signaling and ephrins. Cold Spring Harb Perspect Biol.

[7] Chen J (2012). Regulation of tumor initiation and metastatic progression by Eph receptor tyrosine kinases. Adv Cancer Res.

[8] Muller-Tidow C, Diederichs S, Bulk E, Pohle T, Steffen B, Schwable J, Plewka S, Thomas M, Metzger R, Schneider PM, Brandts CH, Berdel WE, Serve H (2005). Identification of metastasis-associated receptor tyrosine kinases in non-small cell lung cancer. Cancer Res.

[9] Hafner C, Bataille F, Meyer S, Becker B, Roesch A, Landthaler M, Vogt T (2003). Loss of EphB6 expression in metastatic melanoma. Int J Oncol.

[10] Mohamed ER, Noguchi M, Hamed AR, Eldahshoury MZ, Hammady AR, Salem EE, Itoh K (2015). Reduced expression of erythropoietin-producing hepatocyte B6 receptor tyrosine kinase in prostate cancer. Oncol Lett.

[11] Gu Y, Li F, Qian N, Chen X, Wang H, Wang J (2015). Expression of EphB6 in ovarian serous carcinoma is associated with grade, TNM stage and survival. J Clin Pathol.

[12] Liersch-Lohn B, Slavova N, Buhr HJ, Bennani-Baiti IM (2015). Differential protein expression and oncogenic gene network link tyrosine kinase ephrin B4 receptor to aggressive gastric and gastroesophageal junction cancers. Int J Cancer.

[13] Tang XX, Evans AE, Zhao H, Cnaan A, London W, Cohn SL, Brodeur GM, Ikegaki N (1999). High-level expression of EPHB6, EFNB2, and EFNB3 is associated with low tumor stage and high TrkA expression in human neuroblastomas. Clin Cancer Res.

[14] Tang XX, Zhao H, Robinson ME, Cnaan A, London W, Cohn SL, Cheung NK, Brodeur GM, Evans AE, Ikegaki N (2000). Prognostic significance of EPHB6, EFNB2, and EFNB3 expressions in neuroblastoma. Med Pediatr Oncol.

[15] Fox BP, Kandpal RP (2004). Invasiveness of breast carcinoma cells and transcript profile: Eph receptors and ephrin ligands as molecular markers of potential diagnostic and prognostic application. Biochem Biophys Res Commun.

[16] Fox BP, Kandpal RP (2006). Transcriptional silencing of EphB6 receptor tyrosine kinase in invasive breast carcinoma cells and detection of methylated promoter by methylation specific PCR. Biochem Biophys Res Commun.

[17] Yu J, Bulk E, Ji P, Hascher A, Tang M, Metzger R, Marra A, Serve H, Berdel WE, Wiewroth R, Koschmieder S, Muller-Tidow C (2010). The EPHB6 receptor tyrosine kinase is a metastasis suppressor that is frequently silenced by promoter DNA hypermethylation in non-small cell lung cancer. Clin Cancer Res.

[18] Bailey CM, Kulesa PM (2014). Dynamic Interactions between Cancer Cells and the Embryonic Microenvironment Regulate Cell Invasion and Reveal EphB6 as a Metastasis Suppressor. Mol Cancer Res.

[19] Truitt L, Freywald T, DeCoteau J, Sharfe N, Freywald A (2010). The EphB6 receptor cooperates with c-Cbl to regulate the behavior of breast cancer cells. Cancer Res.

[20] Mayer IA, Abramson VG, Lehmann BD, Pietenpol JA (2014). New strategies for triple-negative breast cancer–deciphering the heterogeneity. Clin Cancer Res.

[21] Wang L, Zhao Z, Meyer MB, Saha S, Yu M, Guo A, Wisinski KB, Huang W, Cai W, Pike JW, Yuan M, Ahlquist P, Xu W (2014). CARM1 methylates chromatin remodeling factor BAF155 to enhance tumor progression and metastasis. Cancer Cell.

[22] Zhang K, Corsa CA, Ponik SM, Prior JL, Piwnica-Worms D, Eliceiri KW, Keely PJ, Longmore GD (2013). The collagen receptor discoidin domain receptor 2 stabilizes SNAIL1 to facilitate breast cancer metastasis. Nat Cell Biol.

[23] Boone C, Bussey H, Andrews BJ (2007). Exploring genetic interactions and networks with yeast. Nat Rev Genet.

[24] Hart T, Brown KR, Sircoulomb F, Rottapel R, Moffat J (2014). Measuring error rates in genomic perturbation screens: gold standards for human functional genomics. Mol Syst Biol.

[25] Hosford SR, Miller TW (2014). Clinical potential of novel therapeutic targets in breast cancer: CDK4/6, Src, JAK/STAT, PARP, HDAC, and PI3K/AKT/mTOR pathways. Pharmgenomics Pers Med.

[26] Antonarakis ES, Heath EI, Posadas EM, Yu EY, Harrison MR, Bruce JY, Cho SY, Wilding GE, Fetterly GJ, Hangauer DG, Kwan MF, Dyster LM, Carducci MA (2013). A phase 2 study of KX2–3 91, an oral inhibitor of Src kinase and tubulin polymerization, in men with bone-metastatic castration-resistant prostate cancer. Cancer Chemother Pharmacol.

[27] Martin GS (2001). The hunting of the Src. Nat Rev Mol Cell Biol.

[28] Yu J, Bulk E, Ji P, Hascher A, Koschmieder S, Berdel WE, Muller-Tidow C (2009). The kinase defective EPHB6 receptor tyrosine kinase activates MAP kinase signaling in lung adenocarcinoma. Int J Oncol.

[29] Bhushan L, Tavitian N, Dey D, Tumur Z, Parsa C, Kandpal RP (2014). Modulation of liver-intestine cadherin (Cadherin 17) expression, ERK phosphorylation and WNT signaling in EPHB6 receptor-expressing MDA-MB-231 cells. Cancer Genomics Proteomics.

[30] Prahallad A, Sun C, Huang S, Di Nicolantonio F, Salazar R, Zecchin D, Beijersbergen RL, Bardelli A, Bernards R (2012). Unresponsiveness of colon cancer to BRAF(V600E) inhibition through feedback activation of EGFR. Nature.

[31] Dixon SJ, Costanzo M, Baryshnikova A, Andrews B, Boone C (2009). Systematic mapping of genetic interaction networks. Annu Rev Genet.

