# Supplementary material for: Targeting synthetic lethality between the SRC kinase and the EPHB6 receptor may benefit cancer treatment
Source: Oncotarget. 2016 Jul 13;7(31):50027–42. doi: 10.18632/oncotarget.10569 (PMC5226566; doi:10.18632/oncotarget.10569)
Supplement: Supplementary file 1 [file oncotarget-07-50027-s001.pdf]

Targeting synthetic lethality between the SRC kinase and the EPHB6 receptor may benefit cancer treatment

Supplementary Materials

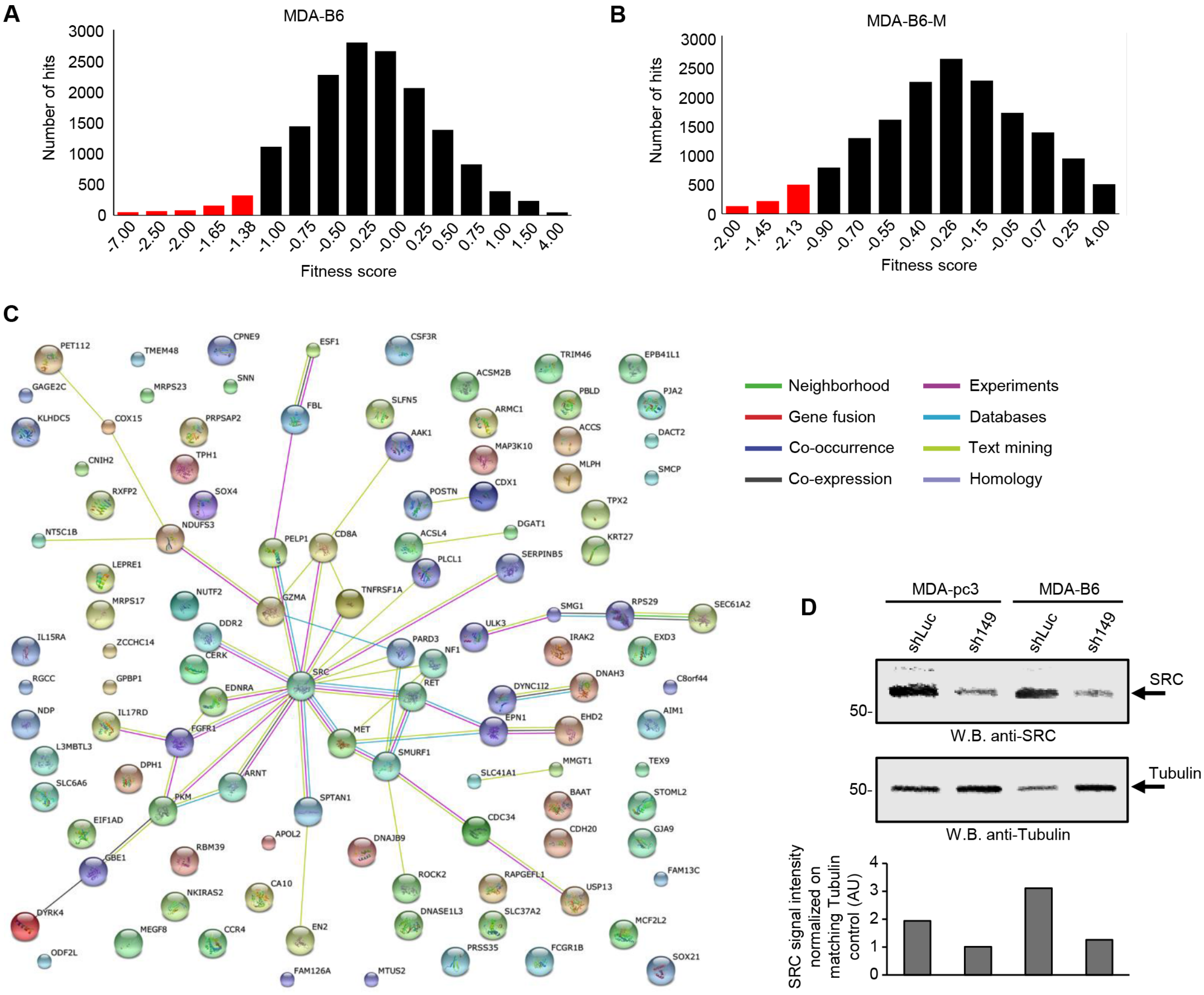

Supplementary Figure S1: Characterization of SL interactions of EPHB6. (**A**) Frequency chart of MDA-B6 DCC scores with *P*-values below 0.05 highlighted in red. (**B**) Frequency chart of MDA-B6-M DCC scores with *P*-values below 0.05 highlighted in red. (**C**) Network generated from the STRING database based on the function interactions of the genes. (**D**) SRC expression in MDA-pc3 and MDA-B6 cells transduced with SRC-targeting shRNA, or non-silencing shLuciferase (shLuc). SRC expression was analyzed by Western blotting with anti-SRC and quantitated by densitometry. SRC quantifications were normalized on matching tubulin controls and presented in arbitrary units (AU).

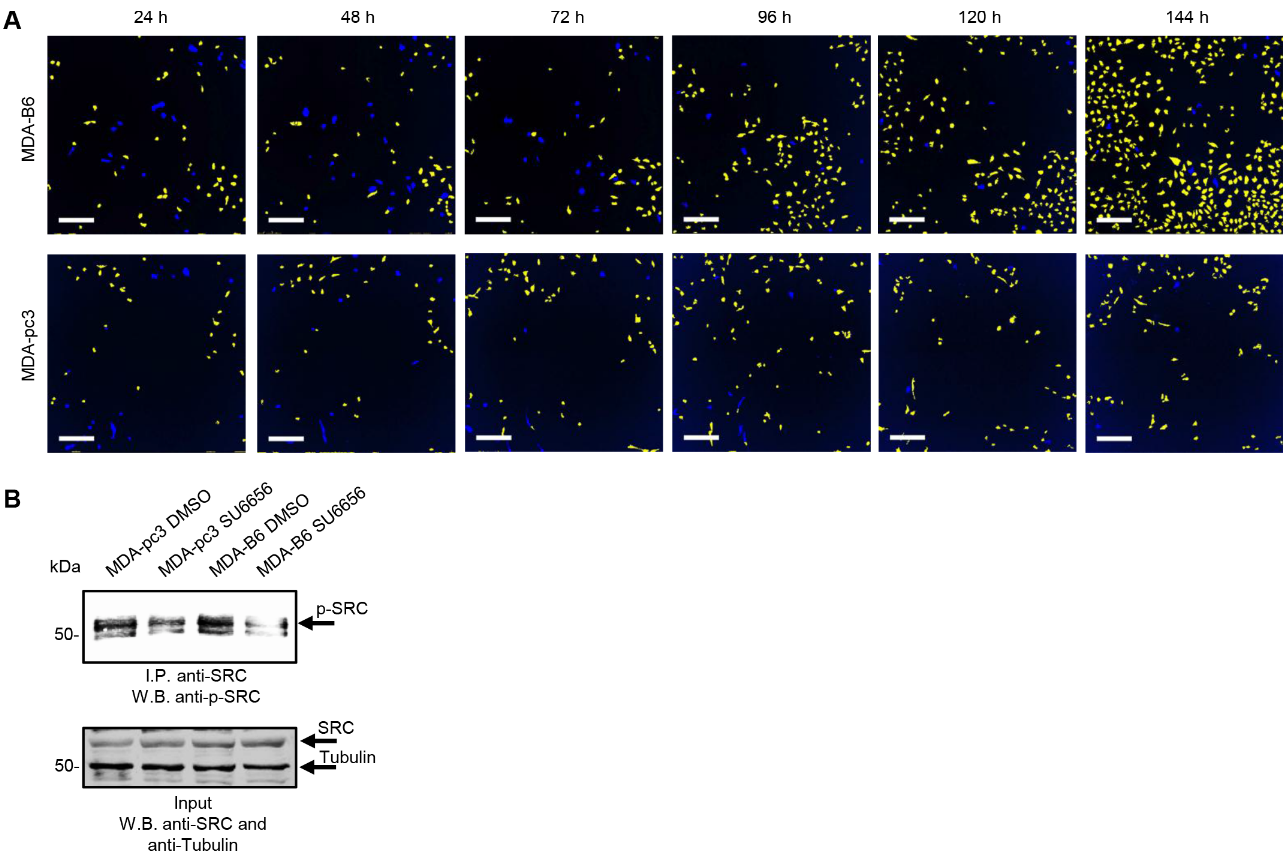

Supplementary Figure S2: Analysis of EPHB6-SRC SL interaction. (**A)** MDA-pc3 and MDA-B6 cells were stably transduced with a *src*-targeting sgRNA construct that also encoded the blue fluorescent protein (BFP) and selected in the presence of 2 µg/ml of puromycin. The selected cells were transiently transfected with Cas9-GFP in 96-well plates. Green and blue fluorescence was quantified using the ImageXpress Micro XLS widefield automated fluorescence microscope and the MetaXpress version 6 software. The figure shows representative images of MDA-pc3 and MDA-B6 cells at consistent locations over the period of six days following Cas9 transfection. Yellow-highlighted cells represent those expressing BFP, while blue-highlighted cells represent those co-expressing BFP and GFP, according to the standard MetaXpress software settings. Scale bar, 250 µM. (**B)** MDA-pc3 and MDA-B6 cells were serum-starved for 24 hours and then treated with 20 µM SU6656 or matching DMSO control for 40 minutes in the presence of 10% FBS. Cells were lysed and immunoprecipitations were performed with anti-SRC. Immunoprecipitates were resolved by SDS-PAGE, transferred to the nitrocellulose membrane and Western blotted with anti-phospho-SRC (anti-p-SRC), recognizing SRC molecules phosphorylated on the activating tyrosine residue. The presence of SRC in matching cell lysates was monitored by Western blotting with anti-SRC.

Supplementary Table S1: List of SL hits from the EphB6 screen

List of EPHB6 synthetic lethal interactions

Gene ID	Gene Symbol	Gene ID	Gene Symbol	Gene ID	Gene Symbol
22848	AAK1	2091	FBL	9584	RBM39
84680	ACCS	2210	FCGR1B	5979	RET
2182	ACSL4	2260	FGFR1	9475	ROCK2
348158	ACSM2B	2574	GAGE2C	6235	RPS29
202	AIM1	2632	GBE1	122042	RXFP2
23780	APOL2	81025	GJA9	55176	SEC61A2
55156	ARMC1	65056	GPBP1	5268	SERPINB5
405	ARNT	3001	GZMA	219855	SLC37A2
570	BAAT	3601	IL15RA	254428	SLC41A1
28984	C13orf15	54756	IL17RD	6533	SLC6A6
56260	C8orf44	3656	IRAK2	162394	SLFN5
56934	CA10	23281	KIAA0774	4184	SMCP
1233	CCR4	57542	KLHDC5	23049	SMG1
925	CD8A	342574	KRT27	57154	SMURF1
997	CDC34	84456	L3MBTL3	8303	SNN
28316	CDH20	64175	LEPRE1	11166	SOX21
1044	CDX1	4294	MAP3K10	6659	SOX4
64781	CERK	23101	MCF2L2	6709	SPTAN1
254263	CNIH2	1954	MEGF8	6714	SRC
1355	COX15	4233	MET	30968	STOML2
1348	COX7AP2	79083	MLPH	374618	TEX9
151835	CPNE9	93380	MMGT1	55706	TMEM48
1441	CSF3R	51373	MRPS17	7132	TNFRSF1A
168002	DACT2	51649	MRPS23	7166	TPH1
4921	DDR2	4693	NDP	22974	TPX2
8694	DGAT1	4722	NDUFS3	80128	TRIM46
55567	DNAH3	4763	NF1	25989	ULK3
4189	DNAJB9	28511	NKIRAS2	8975	USP13
1776	DNASE1L3	93034	NT5C1B	23174	ZCCHC14
1801	DPH1	10204	NUTF2		
1781	DYNC1I2	57489	ODF2L		
8798	DYRK4	56288	PARD3		
1909	EDNRA	64081	PBLD		
30846	EHD2	27043	PELP1		
84285	EIF1AD	5188	PET112L		
2020	EN2	9867	PJA2		
2036	EPB41L1	5315	PKM2		
29924	EPN1	5334	PLCL1		
51575	ESF1	10631	POSTN		
54932	EXD3	5636	PRPSAP2		
84668	FAM126A	167681	PRSS35		
220965	FAM13C	51195	RAPGEFL1		
